# Traditional leadership and the Tokwe-Mukosi induced displacements: Finding the missing link

**DOI:** 10.4102/jamba.v10i1.592

**Published:** 2018-11-15

**Authors:** Kudzayi S. Tarisayi

**Affiliations:** 1 School of Education, University of KwaZulu-Natal, South Africa

## Abstract

There is increasing empirical evidence that the relocation of the victims of the Tokwe-Mukosi floods in Zimbabwe was marred by a combination of challenges. These challenges are argued in this article to have resulted from the adoption of Eurocentric models by government and non-governmental organisation technocrats and experts while relegating traditional leadership and the lived experiences of the displaced to the shadows. The writer provides a summary and critique of the Elizabeth Colson–Thayer Scudder four-stage model and Michael Cernea’s Impoverishment Risks and Reconstruction Model. This article argues that traditional leadership is the missing link in disaster-induced displacement and its integration can overcome most of the challenges faced by the displaced in Zimbabwe. Traditional leadership in Zimbabwe can be traced to precolonial states and it has survived the colonial and postcolonial epochs. The study was guided by the Afrocentric theoretical framework. The case for the integration of traditional leadership was buttressed by numerous arguments. Among these arguments include proximity of traditional leadership to the displaced, the *Zunde raMambo* concept and ubuntu, among others.

## Introduction

The discourse on the adoption rather than adaptation of Western models has lately been met with a vituperative debate from African and decoloniality scholars. The relegation of traditional leadership to the sidelines in efforts to assist displaced persons as revealed by the case of the Tokwe-Mukosi displacements is argued in this article to be because of adoption of Western models. This article starts by tracing the concept of traditional leadership in Zimbabwe. The roles of traditional leadership are also interrogated. The next section presents the Tokwe-Mukosi context. The writer utilises the succeeding section to discuss two selected Western models used in analysing disaster-induced displacements. The theoretical framework guiding this article is discussed in the next section. The discussion of the prolificacy of traditional leadership in disaster-induced displacements in Zimbabwe is preceded by a presentation of the research methodology.

### Traditional leadership in Zimbabwe

The origin of traditional leadership in Africa in general and Zimbabwe in particular can be traced to the precolonial era. Bishi (2015:10) states, ‘the institution of traditional leadership is a product of precolonial, colonial and post-colonial changes’. Hence, this author argues that traditional leadership because of its enduring ability that made it survive the colonial and postcolonial challenges and onslaught on its role should be factored into the relocation of displacement victims and local development. This ability to survive the precolonial, colonial and postcolonial epochs in one form or another makes traditional leadership an important custodian of survival strategies for rural communities, which should not be underestimated in disaster-induced displacements. Traditional leadership in Zimbabwe takes a number of forms. Makahamadze, Grand and Tavuyanago (2009:34) state: ‘The term “chief,” “*ishe*” or “*vashe*” in Shona and “*induna*” in Ndebele, refers to an individual who, by virtue of ancestry, occupies a clearly defined leadership position in an area’. Other titles that are used in Shona for traditional leadership include *Mambo, Sabhuku, Samusha, Sakuvaka* and *Sadunhu* varying with districts around Zimbabwe. The regulatory framework for traditional leaders in Zimbabwe is provided for by various acts of Parliament, the *Chief and Headman Act*, the *Traditional Leaders Act of 1998* and the national constitution. The constitution of Zimbabwe Amendment (No. 20) (2013), Section 280(2) states:

A traditional leader is responsible for performing the cultural, customary and traditional functions of a Chief, head person or village head, as the case may be for his or her community. (p. 110)

Zimbabwe is among the African countries that recognise traditional leadership as revealed by its inclusion in the Zimbabwean constitution under Chapter 15. However, despite this legal cognisance, traditional leadership has been relegated to the background in as far as disaster-induced displacements such as the Tokwe-Mukosi displacements. In ancient times traditional leadership was considered a refuge in terms of disasters such as famine and disease outbreaks, among others.

The role of traditional leadership in Zimbabwe is articulated by the *Traditional Leaders Act of 1998* as well as Section 282 of the constitution of Zimbabwe. Postcolonial governments in Africa initially weakened traditional leadership before realising its important role. Bishi (2015:41) argues that, in Ghana, ‘[*t*]he government realised that traditional leaders were very important in representing traditional and cultural aspects of the community’. By contrast, in Zimbabwe, the government’s realisation only came after the Rukuni Commission, which established that traditional leadership had an important role in Zimbabwe, leading to the enactment of the *Traditional Leaders Act of 1998*. The *Traditional Leaders Act*, Chapter 29:17, reinstated most of the powers of traditional leaders, allocating 23 functions to chiefs as provided under Section 3 of the act.

In addition, Dusing (2001) avers that traditional leadership initially provided socio-economic and political as well as religious functions for local communities. Thus, it follows that playing a more central role in the relocations of displacement victims falls within the socio-economic and political role of the traditional leadership. Additionally, Dlungwana (2002) avers that:

traditional leadership is an institution that is defined by customs and traditions inherited through ancestry that leads a community in a specific area appointed in accordance with the traditions, has authority to rule over people through customary law or an order from government to exercise traditional authority over a tribe. (p. 06)

The Rukuni Commission established that traditional leadership in Zimbabwe has legitimacy in the eyes of the rural communities. Hence, it can be argued that there is acceptancy of the authority of traditional leadership. An integration of traditional leadership in the efforts to assist the displaced fosters acceptancy and a sense of ownership of the decisions made thereof as the rural communities identify with their traditional leadership.

### The context of the Tokwe-Mukosi displacements

Various studies present statistics that indicate the magnitude of displacements worldwide. Cernea and Mathur (2008) state that at least 15 million people each year are displaced from their homes by major development projects globally. Over the last two decades about 280–300 million people have been displaced by development projects worldwide (Ferris 2011). In Africa, the construction of large dams displaced 400 000 people (WCD 2000). Terminski (2013) states that the Akosombo Dam in Ghana displaced about 84 000, the Aswan High Dam in Egypt and Sudan displaced over 120 000 people, the Kossou Dam in Ivory Coast displaced about 85 000 people and the Kariba Dam in Zimbabwe displaced 57 000 people. The recurrence of displacements around the world has led to different classifications of displacement emerging. Displacements around the world have been classified in a number of ways. Classifications that have been utilised in studies include internal displacements (Mooney 2005); climate-induced displacements (Cohen & Bradley 2010); dam-induced displacements (Terminski 2013); development-induced displacement (Smith 2001); and mining-induced displacement (Downing 2003). Conversely, these numerous classifications are by and large linked to the root cause of the displacement. The Tokwe-Mukosi displacements can been classified as disaster-induced displacements. This study conceptualises the Tokwe-Mukosi displacements as disaster-induced displacements as buttressed by the declaration of the floods as a national disaster in February 2014 by the Zimbabwean government (Rusvingo 2014). However, it can also be viewed as internal displacement when regarded from the standpoint of displacements occurring within the confines of the borders of one country. Article 1(k) of the Kampala Convention states (Aspelt & Bradley 2012):

Internally displaced persons are persons or groups of persons who have been forced or obliged to flee or to leave their homes or places of habitual residence, in particular as a result of or in order to avoid the effects of armed conflict, situations of generalized violence, violations of human rights or natural or human-made disasters, and who have not crossed an internationally recognized State border. (p. 01)

Thus, based on the Kampala Convention, the Tokwe-Mukosi displacements can be classified as internal displacements because the movement of the victims was within the borders of Zimbabwe. The definition by the Kampala Convention is also proffered by Article 1(5) of the *Protocol on the Protection and Assistance to Internally Displaced Persons*. In contrast, from another perspective it can be argued that the Tokwe-Mukosi displacements were in actual fact dam-induced displacements as the people were displaced by the floods resulting from the newly constructed Tokwe-Mukosi Dam.

In addition, the Tokwe-Mukosi displacements glaringly exposed the Zimbabwe’s disaster preparedness or lack thereof with arguments that the country has been a victim of displacements in recent years. Hove (2016) argues that the Tokwe-Mukosi flood victims became state victims because of the failure by the Zimbabwean government to provide for the displaced after the occurrence of the disaster. However, the International Peace Institute cited in Chendume (2016:23) contradicts this by tellingly revealing that, ‘[*n*]o country is immune from the forces of nature’. The Zimbabwean government’s approach to assisting people displaced by the Tokwe-Mukosi floods was widely viewed as inadequate (Human Rights Watch 2015; OCHA 2014).

Tokwe-Mukosi Dam is located in Masvingo Province in Zimbabwe. The construction of the dam started in 1998 and was completed in 2016. However, heavy flooding in February 2014 led to the displacement of more than 6000 families (Tarisayi 2014). Tarisayi (2014) reveals that:

the heavy rains and subsequent floods adversely affected 12 villages, explicitly Chekai, Jahwa, Zifunzi, Mharadzano, Chkandigwa and Vhomo in Nemauzhe communal lands; and Tagwirei, Ndove, Matandizvo, Chikosi, Mashenjere and Nongera in Neruvanga communal lands. (p. 166)

The people displaced by the Tokwe-Mukosi Dam were relocated to Chingwizi, Chisase and Masangula (Chendume 2016). Therefore, this author’s thrust is premised within the failure by the government to adequately provide for the victims of the Tokwe-Mukosi floods.

### Western models and their critique

This section of the article briefly abridges two models that inform disaster-induced displacements around the world: the Elizabeth Colson–Thayer Scudder four-stage model and Michael Cernea’s Impoverishment Risks and Reconstruction Model. The author further proffers a critique of Western models based on these two models presented in this article. The Elizabeth Colson–Thayer Scudder four-stage model was formulated in the 1980s. Cernea (1999) notes that the Elizabeth Colson–Thayer Scudder model was primarily utilised in research on voluntary resettlement. However, the Elizabeth Colson–Thayer Scudder model was later applied in cases of involuntary resettlement. The four stages by which persons in sociocultural systems react to resettlement are recruitment, transition, potential development and handing over or incorporation. The model has a number of loopholes. It was developed more than three decades ago and can be critiqued for being antiquated. Additionally, it does not take cognisance of traditional leadership as advocated by this article.

The other Western model informing disaster-induced displacement is Michael Cernea’s Impoverishment Risks and Reconstruction Model. The Impoverishment Risks and Reconstruction Model identifies risks affecting displaced people as ‘landlessness, joblessness, homelessness, marginalization, food insecurity, loss of access to common property resources, increased morbidity and mortality, and community disarticulation’ (Cernea 1999). Cernea’s model is more comprehensive and modern as compared to the Elizabeth Colson–Thayer Scudder model. However, the main weakness of the Impoverishment Risks and Reconstruction Model is neglecting the traditional leadership perspective. Furthermore, neglecting traditional leadership in the Impoverishment Risks and Reconstruction Model arguably stems from it being a Western model. The model does not take into consideration traditional leadership in Africa in general and Zimbabwe in particular.

These models were designed in the West and yielded success in Western countries and even North America. However, they are not necessarily guaranteed to succeed when implemented in Africa because of disparities in context. Hence, the argument by Buntu (2013:2) that Western models are ‘built on a Western premise, filtered through a colonial matrix of power, conceptualised through a racial system of social classification’. Therefore, adaptation of Western models entails a prolongation of the colonial milieu of supremacy, which is skewed against the victims, as in this case of the displaced people. Thus, it is essential that social scientists and practitioners alike in Africa at least adapt Western models to suit the conditions and demands of the continent or alternatively develop a new framework as proposed by this article, which is grounded in the realities of Africa and Africans. This realisation of the inadequacy of accepting Western models as panacea for African development problems in general might have led Mahmood Mandani to argue explicitly (Mwesigire 2016):

I cannot take the design of a Swedish architect to build a house in Uganda. My design must reflect local conditions, use local resources in response to local problems. Anything from the outside must be complementary to this. (n.p.)

Western frameworks are akin to the adaptation of alien architecture, which cannot withstand the vicissitudes of the local realities and environment. The inadequacies of Western frameworks were aptly revealed by the challenges that were glaringly exposed by the relocation of the people displaced by the Tokwe-Mukosi floods in Zimbabwe. Thus this author’s argument that the framework utilised to assist the displaced in Zimbabwe was both irrelevant and not in sync with the realities of the victims of the floods. In addition, the Tokwe-Mukosi floods flagrantly revealed the tendency by African governments in general and the Zimbabwean government in particular to regard Western frameworks and knowledge as panacea for all displacement situations regardless of place and realities of the local population. This article concurs with Robert Chamber’s argument in the article ‘Poverty and livelihoods: Whose reality counts?’ that the realities of the poor should take pre-eminence in any interventions. The reality that matters in displacement scenarios is that of the displaced, not the reality of Eurocentric scholars or technocrats. This argument is further buttressed by Kotze and Kellerman (1997):

The role and status of the technocrat and technocratic approaches contribute not only to the devaluation of indigenous knowledge and experience but also to the side tracking of the people’s emotions and feelings in development. (p. 34)

Hence, the argument by this article that Western approaches despite their perceived strengths relegate the views and lived experiences of the displaced to the background, as shown by the case of the Tokwe-Mukosi displacements in Zimbabwe. Moreover, arguably bookish theories and knowledge can be critiqued for being implemented as one-size-fits-all solutions. This is why African scholar and head of the Archie Mafeje Research Institute for the Social Policy Prof. Sabelo Ndlovu-Gatsheni argues, ‘Africa is today saddled with irrelevant knowledge that disempowers rather than empowers individuals and communities’ (Ndlovu-Gatsheni 2013:11). Thus, it can be reasoned that Western frameworks applied to the Tokwe-Mukosi flood victims instead of ameliorating their plight actually exacerbated their predicament. Therefore, it can be argued that Western knowledge in this case disempowered the displaced Tokwe-Mukosi villagers and compounded their victimhood.

Western frameworks of disaster-induced displacements in general and Cernea’s Impoverished Risks and Reconstruction Model as implemented recently with Tokwe-Mukosi flood victims apparently lack consideration for traditional leadership and its role or lack thereof in the relocation of the displaced. Acquainted with the enervating ramifications of disaster-induced displacements, this article unravels traditional leadership in Zimbabwe and argues that traditional leadership is actually the missing link in mitigating the apparent effects of displacements. Displacements since time immemorial around the world in general and Zimbabwe in particular have had ramifications on people in a multiplicity of facets of their lives. Recent displacements such as the Tokwe-Mukosi disaster have arguably brought to the fore the need to integrate traditional leadership in order to overcome the implications of disaster-induced displacements. The negation of the role of traditional leadership in disaster-induced displacements presents a missing link in displacement discourse as aptly revealed by the Tokwe-Mukosi case study in Zimbabwe. The author submits that traditional leadership is overlooked in displacement discourse because of the universalisation of Euro–North American centric theories and frameworks without due cognisance of the demands and needs of the African context and Africans. Resultantly, there is an acceptance of these ‘alien’ Euro–North American models as silver bullets to address any displacement issues regardless of the structures already in place such as traditional leadership. This critique of alien theories and models is aptly captured by Asante (2007:2), who submits ‘a severe criticism over the preponderant Eurocentric myths of universalism and challenges the colonizing concepts and racist theories that preside over the triumph of Western thought’. The shortcomings of the Eurocentric model is further explicitly revealed by Mbembe (2016):

A critique of the dominant Eurocentric academic model – the fight against what Latin Americans in particular call ‘epistemic coloniality’, that is the endless production of theories that are based on European traditions. These are produced nearly always by Europeans or Euro-American men who are the only ones accepted as capable of reaching universality; they involve a particular anthropological knowledge, which is a process of knowing about Others – but a process that never fully acknowledges those Others as thinking and knowledge producing subjects. (p. 36)

It is against this background of the criticisms noted of Eurocentric models that this article argues for the inclusion of traditional leadership in Zimbabwe.

A substantial amount of literature has been published on the Tokwe-Mukosi disaster and its ensuing displacements. These studies have rallied around a number of standpoints; among the most prominent perspectives that have emerged include the human rights perspective (Chendume 2016; Nyamafufu 2014); political perspective (Hove 2016; Mtimba 2014 cited in Hove 2016; Tarisayi 2015) and a livelihoods perspective (Mutangi & Mutari 2014; Rusvingo 2014; Tarisayi 2014). However, the aim of this treatise is to argue that Western frameworks have weaknesses, to proffer the role that can be played by traditional leadership in Zimbabwe in mitigating the effects of disaster-induced displacements and to further enunciate that traditional leadership is in actual fact the missing link in the disaster-induced displacements discourse in Africa. The argument pursued by this article leans towards Afrocentric theoretical grounding to a greater extent.

### Theoretical framework

As Imenda (2014:189) reveals, ‘a theoretical framework refers to the theory that a researcher chooses to guide him/her in his/her research’. Thus, it is against this background that this study was guided by the Afrocentric theoretical framework. Chawane (2016) summarises Afrocentric theory as an approach that:

proposes that blacks (at home and abroad) must look at knowledge from an African perspective. It suggests looking at matters at hand from an African viewpoint; that we misunderstand Africa when we use viewpoints and terms other than that of the African to study Africa. (p. 78)

Thus, disaster-induced displacements in Africa should be viewed from an African perspective according to the Afrocentric theory. Asante (2009:04) avers that ‘Afrocentric theory seeks neither a totalizing nor a universal scope and certainly not an essentialized perspective on knowledge’. In addition (Asante 2009):

as a cultural theory Afrocentricity is committed to the reclamation of ancient African classical civilizations as the place for interpreting and understanding the history of African peoples, narratives, myths, spirituality, and cosmogonies. (p. 4)

Therefore, this study is premised on the need to reclaim the role of traditional leadership in the relocation of the displaced in Zimbabwe. The welfare of the displaced is best catered for by understanding the existing safety nets such as *Zunde* and the integration of traditional leadership. This academic work identifies with Asante (1998:xi), who argues ‘the principal motive behind [*Afrocentric*] intellectual work seems to have been the use of knowledge for the cultural, social, political, and economic transformation of African people’. Hence, the author utilises the shortcomings of the Western models reviewed in this article to argue for the role of traditional leadership to a greater extent.

## Research methodology

This article utilises a case study of the Tokwe-Mukosi displacements to argue for the incorporation of traditional leadership in disaster-induced displacements. Stake (2000:435) states that case studies are ‘one of the most common ways to do qualitative inquiry’. Additionally, Yin (2003:02) opines that ‘the distinctive need for case studies arises out of the desire to understand complex social phenomena’ such as disaster-induced displacements. The article uses a sample of two Western models that were applied in the African context. The researcher reviewed studies and articles published on traditional leadership in Zimbabwe, Western models and Afrocentric theoretical framework.

### The fecundity of traditional leadership

#### Proximity of traditional leadership to the displacement victims

The argument for the role of traditional leadership in relocating the displaced (as was the case in the Tokwe-Mukosi floods) is premised on the traditional leadership’s proximity to the victims. Traditional leadership in its various forms live among the victims of displacement. The Tokwe-Mukosi floods mainly affected 12 villages under two chiefs (Tarisayi 2014). Thus, it can be revealed from the onset that efforts to assist the victims should have started with consulting the 12 village heads and 2 chiefs, who shared the realities of flood victimhood as they were in the same situation with their fellow villagers. Instead of relying on the Eurocentric models and the expertise of technocrats who are far removed from the realities of the affected, consulting the local traditional leaders would have assisted in addressing some of the challenges that came with being displaced. As the minister of local government in the Zimbabwean government revealed, ‘traditional leaders were linchpins in the community development machinery as they were closest to [*the*] centre of activity where they were best placed to effectively interpret people’s views and aspirations’ (*The Manica Post*, 03 August 2003). Therefore, it can be reasoned that any efforts to assist the displaced in Zimbabwe should involve the traditional leadership that is closest to the victims. In the case of the Tokwe-Mukosi victims, village heads such as Chekai, Jahwa, Zifunzi, Mharadzano, Chkandigwa and Vhomo should have been empowered to play a leading role in assisting the victims in their villages. The author argues that the lived experiences of the village heads and chiefs should be at the centre of any interventions as they are closer to the victims than the government and Non-Governmental Organisation (NGO) personnel who descend on the area after disaster strikes.

#### Involvement of traditional leadership as decentralisation

The transfer of the role to mitigate disasters to traditional leadership can also be viewed as ascribing to the dictates of decentralisation. Kotze (1997) avers that the concept of decentralisation subsumes concepts such as devolution, deconcentration, delegation and privatisation. The daunting task of relocating the displaced during the Tokwe-Mukosi national disaster was delegated to a national and provincial taskforce team, which was far removed from the lived experiences and realities of the displaced. The displaced were not represented and neither were their views; thus questionable decisions were made, all under the guise of resource constraints. This centralisation of decision-making as revealed by the role played by the provincial taskforce in the Tokwe-Mukosi displacements is critiqued by Kotze (1997:33), who reasons: ‘through over centralisation decisions on simple and straightforward matters took a long time’. Decentralisation of logistical arrangements to assist the displaced to traditional leadership helps mitigate the challenges endured by the displaced people. Traditional leaders live among the people who are affected by the displaced and, more, the displaced are their kith and kin. Therefore, the traditional leadership can be argued to be in sync with the realities of the displaced as well as local problems and needs. Hence, the government and NGOs should empower the traditional leadership to be in a position to take up the leadership role in disaster management in their communities.

### The ***Zunde raMambo*** concept

The integration of traditional leadership in the relocation of the Tokwe-Mukosi victims could have facilitated access to the *Zunde raMambo*. The *Zunde raMambo* concept entails ‘plenty of grain stored for future use by people in a particular community’ (Mararike 2000:94). Mushishi (2010) adds:

The *zunde ra mambo* cultural concept refers to a tribal practice where all people under one chief take turns to till portions of the chief’s field as a way of preparing crops whose harvest would be used in times of drought. (p. 133)

In addition, Mandizadza, Bhatasara and Nyamwanza (2014) state:

*Zunde* was meant to be a ‘safety net’ that guaranteed relief to the needy in the community during times of hardships and calamity and it was generally practised as a powerful ritual in rural community organisations, arousing cohesive emotions. (p. 209)

The *Zunde* concept is widespread among the Shona communities in Zimbabwe and therefore tellingly provides a safety net that can be accessed in times of calamity such as disaster-induced displacements. Eurocentric models reviewed in this article fail to take due cognisance of the African safety nets such as *Zunde*, which can play an immense role in ameliorating the challenges faced by the displaced. Therefore, it can be argued that the two chiefs whose communities were affected by the Tokwe-Mukosi floods could have utilised their *Zunde* granary to feed the affected villagers during their distress. In addition, the two concerned chiefs could have extended the *Zunde raMambo* concept to the construction and provision of accommodation to the displaced Tokwe-Mukosi people.

However, a word of caution is necessary on the role that can be played by traditional leadership in ensuring that the uprooted victims of disaster are well catered for pertaining to conditions that should be in place to ensure positive results. The benefits of the role of the traditional leadership can only be made possible in communities that have embraced the concept of *Zunde raMambo*. The *Zunde raMambo* concept will ensure that the victims have adequate food as well as accommodation. Moreover, such communities have a history of cooperation and it is easier to harness their networks to conglomerate and cleave in providing for the victims of the disaster.

### Traditional leadership as upholders of ubuntu

Integration of traditional leadership can facilitate the ubuntu benefits to be reaped by the displacement victims. Traditional leaders in Zimbabwe as in other Southern African countries are regarded as upholders of the African philosophy of ubuntu. Ubuntu can be argued to be mainly evident in rural areas in Zimbabwe because of the role of traditional leaders. Chakunda and Chikerema (2014:72) argue that ‘there is growing recognition that African communities being mostly rural, continue to place high value on indigenous customs and tradition in their day to day lives’. Rural areas therefore can be reasoned to be fortresses of ubuntu and hence the need to integrate traditional leadership in order to help disaster-induced displacement victims.

The concept of ubuntu has been interrogated and applied in various fields of study by numerous scholars. Archbishop Tutu in Hailey (2008:2) reflects that ubuntu is ‘the essence of being human, and that it is part of the gift that Africa will give the world’. Moreover, Khoza (2005) avers ‘an individual’s humanity is expressed through a person’s relationship with others and theirs in turn through recognition of the individual’s humanity’. In addition, Van Stem (2016) adds: ‘Ubuntu considers the needs of the group first, believing that in so doing, individual needs and desires will be met’. Any intervention should be guided by the African philosophy of ubuntu, factoring in traditional leadership and the victims’ needs.

In addition, in advocating for the role of ubuntu, Metz (2015:03) reasons that ‘indigenous Southern African peoples by and large sought to live genuinely human lives, or at least adhered to worldviews prizing that’. The challenges that were faced by the victims of the Tokwe-Mukosi floods related to Chingwizi can be viewed as results of the negation of ubuntu. Mangaliso and Mzamo (2001:24) observe that ‘neglecting Ubuntu can be a source of disharmony and strife in the daily-lived, African environment’. The challenges faced by the displaced at Chingwizi such as rampant sexual abuse of women and girls (Chendume 2016; Tarisayi 2014) are attributable to the neglect of ubuntu as observed by Mangaliso and Mzamo in a study in South Africa. Hence, the author’s argument for the integration of traditional leadership in disaster-induced displacements to avert the disharmony witnessed at Chingwizi. As already revealed by this article, traditional leadership is an embodiment of ubuntu and therefore its integration would overcome some of the challenges encountered at Chingwizi.

## Conclusion

From the foregoing, it can be contended that there is indeed a compelling argument for traditional leadership as the missing link in disaster-induced displacements in Zimbabwe. This article gave a review and critique of Western models that guided the relocation of people following the Tokwe-Mukosi disaster in Zimbabwe. In addition, weaknesses of Eurocentric and Euro–North American models were also revealed by this author and hence the article’s advocacy for the centrality of traditional leadership as the missing link in disaster-induced displacements. Traditional leadership was argued by the researcher as the missing link in the Tokwe-Mukosi displacement discourse. Guided by an Afrocentric theoretical framework, the article argued that traditional leadership is better positioned to assist victims of disaster displacements. Traditional leaders are in proximity to the displacement victims, which is instrumental for assisting displacement victims. In addition, traditional leaders in Zimbabwe are the custodians of ubuntu and the *Zunde raMambo*. Both ubuntu and the *Zunde raMambo* were argued to be essential in addressing the challenges faced by disaster-induced displacement victims. The role of traditional leadership has been aptly revealed by the strengths that were enumerated in this article.
